# The relative change in regulatory T cells / T helper lymphocytes ratio as parameter for prediction of therapy efficacy in metastatic colorectal cancer patients

**DOI:** 10.18632/oncotarget.22606

**Published:** 2017-11-21

**Authors:** Tong Xu, Jiezhen Lu, Hanxiang An

**Affiliations:** ^1^ Department of Medical Oncology, Xiamen Cancer Hospital, The First Affiliated Hospital of Xiamen University, Xiamen, Fujian, China

**Keywords:** colorectal cancer, regulatory T cells, therapy efficacy, T helper lymphocytes, change

## Abstract

**Purpose:**

The evaluation of regulatory T (Treg) (CD4^+^CD25^high^ CD127^neg^) lymphocyte count with respect to the T helper (TH) (CD4) number has been shown to represent the main immune parameters capable of signifying the functional status of the anticancer immunity in cancer patients. This study is aimed to explore a correlation between therapy efficacy and changes in Treg/TH ratio and other biochemical and haematological parameters in patients with metastatic colorectal cancer (mCRC).

**Experimental Design:**

Measurements of regulatory T cells were performed by flow cytometric analysis pre- and post-therapies in a prospective study.

**Results:**

We investigated levels of Treg/TH ratio in the peripheral blood of 25 mCRC patients pre- and post-chemotherapy ± targeted therapy. There were significant differences in levels of Treg/TH ratio pre- and post-treatments among patients on study, patients with partial response (PR), stable disease (SD) and progressive disease (PD) (*P*= 0.012, *P*= 0.011, and *P*= 0.043, respectively). Moreover, the relative change in Treg/TH ratio showed statistically significant difference among patients with PD as compared to those with PR and SD. Our findings demonstrated a statistically significant strong correlation between the relative change in Treg/TH ratio and therapeutic response. (Spearman's rho= 0.788/*p*<0.001).

**Conclusions:**

The monitoring of the relative change in Treg/TH ratio could constitute a promising clinical index for response prediction and a timely change in regimen. Further prospective evaluations of these parameters investigated, particularly their association with overall survival, are warranted.

## INTRODUCTION

Colorectal cancer (CRC) is the third most commonly diagnosed cancer and the fourth major reason for tumor-associated deaths worldwide [1]. The present treatment choice for the late CRC is represented by combination chemotherapy with fluorouracil plus leucovorin together with oxaliplatin (FOLFOX) or CPT-11 (FOLFIRI) given alone, or in combination with cetuximab or bevacizumab, monoclonal antibodies that target the epidermal growth factor receptor or the vascular endothelial growth factor, respectively. The effect of these combination therapies on peripheral immune responses is currently rather poorly explored. In addition, the putative alteration of immune parameters by therapy also remains basically unknown.

Immunosuppressive cells and molecules are key issues that potentially abolish the efficacy of established anticancer immunotherapies. These immunosuppressive elements are likewise ample in the microenvironment of tumor and in the blood circulation of tumor sufferers [2]. Regulatory T cells (i.e. Tregs) have been implicated as one such immunosuppressive entity through preclinical and clinical observations. In this process, the increased levels of functional Tregs can play an important role [3, 4]. In cancers, Tregs are able to hinder the development of antitumor immunity and favor the creation of the immunosuppressive tumor microenvironment (TME), which contributes to tumor escape and cancer progression [5].

Tregs exert their function of immune suppression through both cell contact-dependent and -independent mechanisms. First, Tregs secrete immune suppressive cytokines containing IL-10, IL-35, and TGF-β to keep immune responses in check. Second, Tregs target effector T cells or APCs for killing by secreting granzyme B. Third, Tregs inhibit effector T cell and APC activity by their cell-surface bound CTLA-4 and LAG3. Lastly, Tregs disrupt effector T cell metabolism through the delivery of cyclic adenosine [6].

As demonstrated by recent advances in human vaccines and immune-therapeutics, adaptive immune responses can control tumor growth. However, immune regulatory networks suppress anti-tumor immune responses and thus are less effective for immunotherapies. A large body of data now associates increased frequencies of Tregs with various types of tumors [7]. In human colon cancer, many researches have showed increased CD4^+^CD25^high^ FoxP3^**+**^ Treg cells in peripheral blood mononuclear cells (PBMCs) and draining lymph nodes, which can suppress antigen-specific CD4^+^ T cells. Surgical resection of colorectal carcinoma serves to reduce the Treg cell number and restore antigen-specific CD4^+^ activity [8, 9]. Pre-clinical murine models have demonstrated that metastatic spread of ErbB2-transformed mammary carcinoma cells was dependent on Tregs, whose major pro-metastatic function appeared to be nuclear factor kappa-B ligand (RANKL) production [10].

While immune function status considerably influences the prognosis of the majority of cancer patients, including digestive tract cancer sufferers [11], the host-mediated antitumor defense response is linked to rearrangements of different hematological constituents, like leukocyte, particularly the neutrophils, lymphocytes and monocytes. Moreover, alterations in blood count values reflect the dynamic balance between anticancer and tumor-promoting functions of the immune system. More recent evidence has demonstrated that variables derived from blood routine parameters, i.e. platelet-lymphocyte ratio (PLR) and neutrophil-lymphocyte ratio (NLR), could do duty for a simple indicator of the immune function and might have prognostic value in solid cancer sufferers [11]. The systemic immune-inflammation index (SII) has been reported lately as a prognostic indicator in some malignant tumors including hepatocellular carcinoma, lung carcinoma and colorectal cancer [12–14]. Compared with other potential markers, these indices boast the superiority of being economical and reproducible. Among these, NLR and PLR stand for the most usual indicators as markers of chemotherapy response [15, 16]. An elevated NLR is related to unfavorable prognosis and poor response to therapies [17, 18].

Kitayama et al reported that low NLR is associated with an improved disease control and oxaliplatin response in unresectable mCRC patients [19]. In agreement with these studies, Wu et al showed that tumor sufferers with a low NLR benefit more from oxaliplatin-based first-line treatment, and that tumor sufferers with a sharp decline in NLR after 1 cycle of chemical therapy likewise reveal improved disease control and progression-free survival (PFS), as its decrease may show the good response to chemical therapy. Furthermore, they observed the same results with respect to PLR [19].

So far cancer biomarkers, namely CA199, CA724, CA125 and CEA, have been usually utilized for assessing clinical efficacy and prognosis in cancer sufferers. Both tumor biomarkers and haematological variables have been investigated as predictor for the sensitivity of chemical therapy and prognosis [19].

Therefore, the purpose of our study is to find out whether changes in Treg/TH ratio and other haematological parameters can be used to monitor response to treatment in metastatic colorectal cancer (mCRC) patients, in addition to standard clinical and radiological evaluation. The parameters investigated in the study are evaluated with regard to predictive potential for response to treatment.

## RESULTS

### Patient characteristics

A total of 25 patients with stage IV colorectal carcinoma undergoing classical anticancer therapies were enrolled in this study. Cancer sufferer characteristics are reported in Table 1, covering clinical efficacy, age, gender, ECOG PS, smoking and alcohol-drinking history, first-degree relative cancer history, differentiation grade, stage, tumor location, differentiation grade, KRAS status and chemotherapy regimens and response to treatment. The median patient age was 59 years (ranging 33–74 years), and the male-female ratio was 16:9. Their lesions were all identified as glandular cancer. Of the 25 cancers analysed, 72% were in the colon, while 28% were in the rectum. Regarding chemotherapy regimens, 4 patients (16%) received FOLFOX6, 7 (28%) received FOLFIRI, 10 patients (40%) received chemotherapy plus bevacizumab, and 4 patients (16%) received chemotherapy plus Cetuximab combination. Nine of them received a first-line therapy, while 10 and 6 received a second-line therapy and a third-line therapy, respectively. There were no patients who had a complete response, and 8, 12 and 5 cancer sufferers had PR, SD and PD, respectively at evaluation. With regard to laboratory data, the median (IQR) values of blood count parameters are revealed to be normal. Other biochemical parameters, such as ALP and LDH levels, appear normal in the majority of cancer sufferers. The median (IQR) values of serum level of CA50, CA125, CA199, CA724 and CEA are shown at the normal range.

**Table 1:** Patient demographics and clinical characteristics

Response to treatment	PR	SD	PD	total
number	8	12	5	25
Age(years)	65(33-74)	57(43-70)	62(41-72)	59(33-74)
<60	25%	75%	52%	52%
≧60	75%	25%	48%	48%
Gender				
Male	4(50%)	7(58.3%)	0(0%)	16(64%)
Female	4(50%)	5(41.7%)	5(100%)	9(36%)
ECOG PS				
0	3(37.5%)	7(58.3%)	2(40%)	12(48%)
1	5(62.5%)	5(41.7%)	3(60%)	13(52%)
2	0(0%)	0(0%)	0(0%)	0(0%)
Smoking history				
No	6(75%)	10(83.3%)	5(100%)	21(84%)
Yes	2(25%)	2(16.7%)	0(0%)	4(16%)
Alcohol-drinking history				
No	7(87.5%)	10(83.3%)	5(100%)	22(88%)
Yes	1(12.5%)	2(16.7%)	0(0%)	3(12%)
First-degree relative cancer history				
No	8(100%)	10(83.3%)	5(100%)	22(88%)
Yes	0(0%)	2(16.7%)	0(0%)	3(12%)
Stages				
IVA	4(50%)	3(25%)	3(60%)	10(40%)
IVB	3(37.5%)	6(50%)	1(20%)	10(40%)
IVC	1(12.5%)	3(25%)	1(20%)	5(20%)
Tumor localization				
Ascending colon	1(12.5%)	4(33.3%)	1(20%)	6(24%)
Transverse colon	1(12.5%)	0(0%)	0(0%)	1(4%)
Descending colon	1(12.5%)	0(0%)	2(40%)	3(12%)
Sigmoid colon	3(37.5%)	4(33.3%)	1(20%)	8(32%)
Rectum	2(25%)	4(33.3%)	1(20%)	7(28%)
Differentiation grade				
1	0(0%)	1(8.3%)	0(0%)	1(4%)
2	7(87.5%)	7(58.3%)	3(60%)	17(68%)
3	1(12.5%)	3(33.3%)	2(40%)	7(28%)
KRAS status				
Wild type	2(25%)	3(25%)	1(20%)	6(24%)
Mutated	1(12.5%)	2(16.7%)	2(40%)	5(20%)
Missing	5(62.5%)	7(58.3%)	2(40%)	14(56%)
Line of therapy				
First	5(62.5%)	2(16.7%)	2(40%)	9(36%)
Second	2(25%)	7(58.3%)	1(20%)	10(40%)
Third	1(12.5%)	3(25%)	2(40%)	6(24%)
Associated therapy				
FOLFOX6	2(25%)	2(16.7%)	0(0%)	4(16%)
FOLFIRI	1(12.5%)	4(33.3%)	2(40%)	7(28%)
CT+B	3(37.5%)	5(41.7%)	2(40%)	10(40%)
CT+Cetuximab	2(25%)	1(8.3%)	1(20%)	4(16%)
Neutrophils×103/mm3	3.26±1.70	3.24±1.31	4.02±0.85	3.40±0.27
Lymphocytes×103/mm3	1.36±0.39	1.38±0.62	1.68±0.78	1.43±0.12
Platelets×103/mm3	297±150	249±99	251±59	264±22
Hb(g/L)	118±16	133±15	132±14	128±3
Albumin(g/L)	41.2±4.0	44.8±3.1	45.4±1.6	43.8±0.7
LDH(IU/L)	318(150-533)	222(161-1722)	471(131-654)	251(161-1722)
ALP(U/L)	101(50-130)	100(54-378)	115(89-187)	106(50-378)
CA50(U/mL)	21.7(5.7-57.4)	13.2(0.5-199.7)	53.2(22.7-500)	24.2(0.5-500)
CA125(U/mL)	7.4(2.7-18.2)	7.6(2.0-24.7)	46.4(11.8-99.1)	9.7(2.0-99.1)
CA199(U/mL)	25.6(8.3-139.9)	16.2(1-1404.8)	113.1(28.2-4095.1)	28.2(1-4095.1)
CA724(U/mL)	3.2(1.0-18.6)	4.4(1.0-1249)	7.5(5.8-30.3)	5.6(1.0-1249)
CEA(ng/mL)	2.5(0.3-81.5)	4.3(0.5-396.2)	72.3(9.8-299.6)	4.7(0.3-396.2)

The median and frequency (%) or interquartile range (IQR) for all parameters are shown. ECOG, Eastern Cooperative Oncology Group; PS, performance status; CT, chemotherapy; B, bevacizumab; Hb, hemoglobin; CEA, carcinoembryonic antigen; ALP, alkaline phosphatase; LDH, lactate dehydrogenase; CEA, carcinoembryonic antigen; PR: Partial response; SD: Stable disease; PD: Progression disease.

### Treg/TH ratio levels in peripheral blood

**Figure 1:**
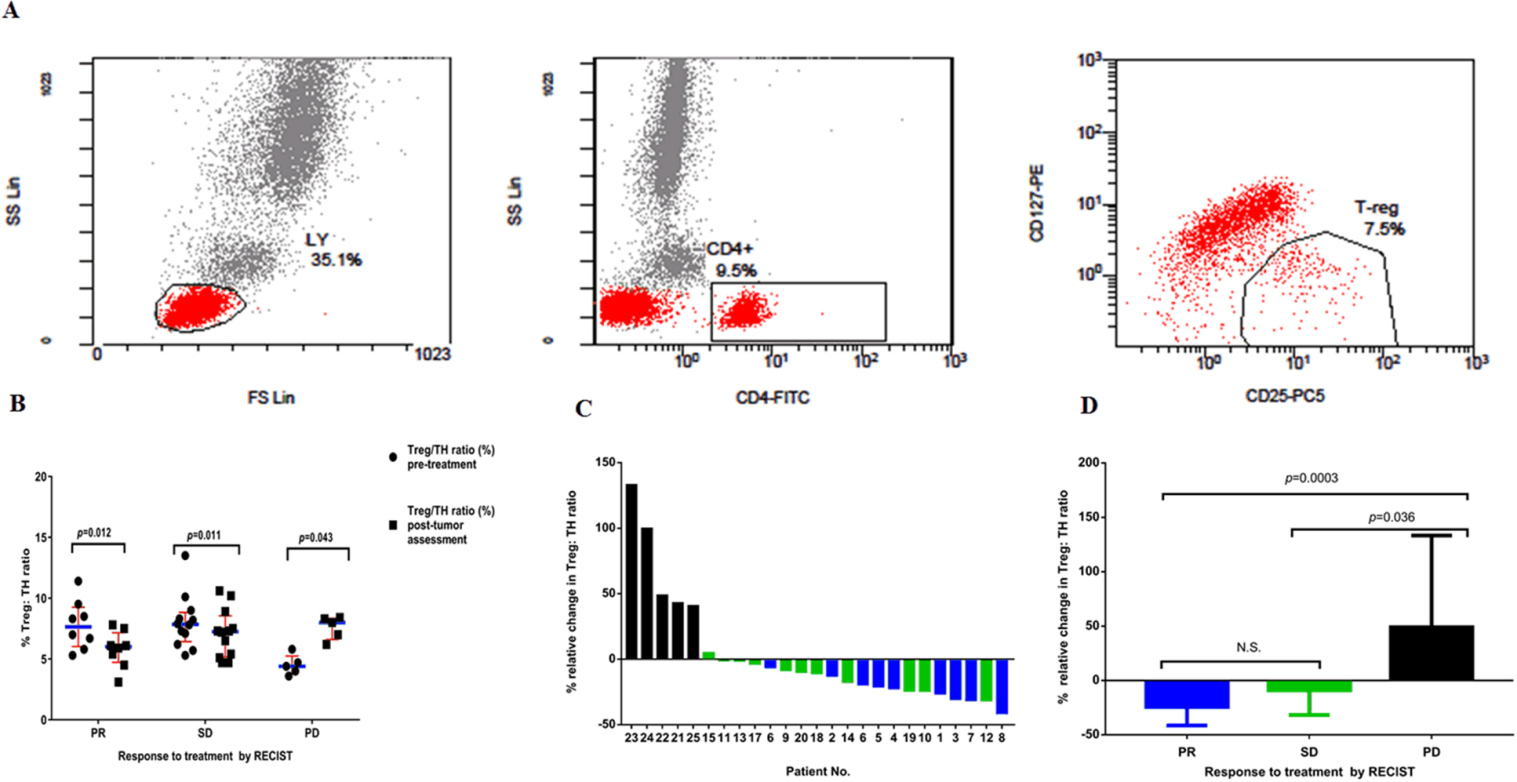
**(A)** Expression of CD25^+^CD127^neg^ in T helper lymphocytes (CD4^+^). PBMCs from a patient were analyzed by flow cytometry after cell surface staining with FITC-conjugated anti-CD4, PE-conjugated anti-CD127, PCy5-conjugated anti-CD25. **(B)** Percentage of CD4^+^CD25^+^CD127^neg^ Tregs in TH population (CD4^+^) pre-treatments and post-tumor assessment in the peripheral blood of all patients enrolled on study. Results are divided in six dot plots, representative of the levels of Treg/TH ratio pre-treatments versus post-tumor assessment in patients who showed PR, SD and PD. *P* values are the result of the nonparametric Wilcoxon Signed Rank test. In all dot plots, the median and interquartile range are shown. **(C)** The relative changes in the ratio of Treg/TH pre-treatments versus post-tumor assessment in patients with colorectal cancer. Waterfall plot of the relative change in the ratio between regulatory T cells and T helper lymphocytes (CD4^+^) (Treg/TH ratio) in the course of therapy in patients (n=25) with metastatic colorectal cancer. Each column represents a case, and the color indicates best overall response for treatment. Blue, PR; green, SD; black, PD. **(D)** Results are divided in three histograms, representative of the relative change of Treg/TH ratio in patients who showed PR, SD and PD. Values are the median (interquartile range). Nonparametric Kruskal–Wallis test was used to determine statistical difference between groups. Tregs, regulatory T cells; RECIST, Response Evaluation Criteria in Solid Tumors; PR, partial response; SD, stable disease; PD, progressive disease. *P* values<0.05 are considered as statistically significant. N.S.: non-significant.

We investigated levels of the Treg/TH ratio in the peripheral blood of 25 mCRC sufferers’ pre- and post-treatments. Tregs are defined as the CD4^+^CD25^+^CD127^neg^ population (Figure 1A). We correlated the change of Treg/TH ratio levels with anticancer response to treatment by RECIST (Response Evaluation Criteria in Solid Tumors) criteria. We observed that 8/25 patients showed PR by RECIST, 12/25 patients had SD, and 5/25 had PD (Table 1). There were distinct changes in levels of Treg/TH ratio pre- and post-treatments among cancer sufferers with PR, SD and PD (*P*=0.012, *P*=0.011, and *P*=0.043, respectively) (Figure 1B). The three groups were compared: PR versus SD versus PD cancer sufferers. A waterfall plot of the relative variation in the Treg/TH ratio in the therapeutic process is shown in Figure 1C. The assessment of the relative change in the Treg/TH ratio revealed statistically significant difference between patients with PD and those with PR and SD (Figure 1D).

### Relationship between therapy efficacy and changes of hematologic parameters

**Figure 2:**
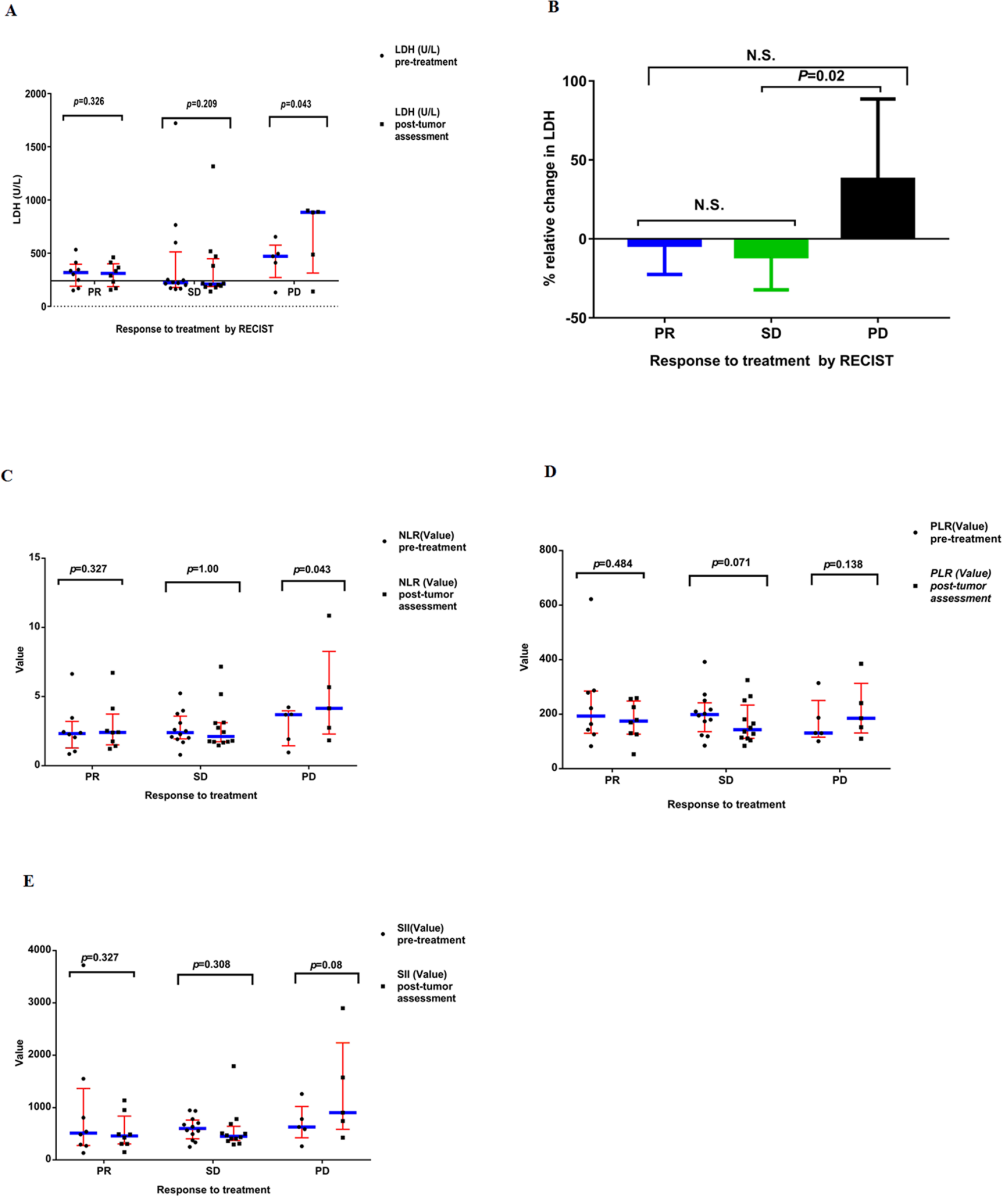
**(A)** Serum levels of LDH pre-treatments and post-tumor assessment in the all patients enrolled on study. Results are divided in six dot plots, representative of the levels of LDH pre-treatments versus post-tumor assessment in patients who showed PR, SD and PD. *P* values are the result of the nonparametric Wilcoxon Signed Rank test. The horizontal line of X axis represents the upper limit of normal (ULN) value of LDH. **(B)** The relative changes in the value of LDH pre-treatments versus post-tumor assessment in patients with colorectal cancer. Results are divided in three histograms, representative of the relative change of LDH in patients who showed PR, SD and PD. Values are the median (interquartile range). Nonparametric Kruskal–Wallis test was used to determine statistical difference between groups. **(C)** The value of NLR pre-treatments and post-tumor assessment in the all patients enrolled on study. Results are divided in six dot plots, representative of the value of NLR pre-treatments versus post-tumor assessment in patients who showed PR, SD and PD. *P* values are the result of the nonparametric Wilcoxon Signed Rank test. **(D)** The value of PLR pre-treatments and post-tumor assessment in the all patients enrolled on study. Results are divided in six dot plots, representative of the value of PLR pre-treatments versus post-tumor assessment in patients who showed PR, SD and PD. *P* values are the result of the nonparametric Wilcoxon Signed Rank test. **(E)** The value of SII pre-treatments and post-tumor assessment in the all patients enrolled on study. Results are divided in six dot plots, representative of the value of SII pre-treatments versus post-tumor assessment in patients who showed PR, SD and PD. *P* values are the result of the nonparametric Wilcoxon Signed Rank test. In all dot plots, the median and interquartile range are shown. LDH, lactate dehydrogenase; RECIST, Response Evaluation Criteria in Solid Tumors; PR, partial response; SD, stable disease; PD, progressive disease. NLR, neutrophil-to-lymphocyte ratio; PLR, platelet-lymphocyte ratio; SII, systemic immune-inflammation index; RECIST, Response Evaluation Criteria in Solid Tumors. *P* values<0.05 are considered as statistically significant. N.S.: non-significant.

We correlated the change of hematologic parameters including LDH, NLR, PLR and SII with anticancer response to treatment by RECIST criteria. There was a remarkable rise in levels of LDH after therapy in the patients with PD (*P*=0.043) with regard to the values found prior to therapy, whereas it decreased (non-significantly) in the tumor sufferers with PR and SD (*P*=0.326, and *P*=0.209, respectively) (Figure 2A). Again the three groups were compared: PR versus SD versus PD patients. The assessment of the relative change in the LDH levels did not reveal any statistically marked variation between tumor sufferers with PR and those with SD and PD. However, our results showed that a marked variation was observed between tumor sufferers with SD and those with PD (Figure 2B). We correlated the changes ofNLR, PLR and SII with their anticancer responses to treatment by RECIST criteria, respectively. There was a marked rise in NLR after therapy in the patients with PD (*P*=0.043) with regard to the values found prior to therapy, whereas our results revealed that no obvious variation was observed in NLR pre- and post-treatments in the tumor sufferers with PR and SD (*P*=0.327, and *P*=1.00, respectively) (Figure 2C). There was an increase (non-significant) in the value of PLR after therapy in the patients with PD (*P*=0.138) with regard to the values found prior to therapy, whereas it decreased (non-significantly) in the patients with PR and SD (*P*=0.484, and *P*=0.071, correspondingly) (Figure 2D). Our results showed that an increase (non-significant) was observed in the value of SII after therapy in the patients with PD (*P* = 0.08) with respect to the values found prior to therapy, whereas it decreased (non-significantly) in the tumor sufferers with PR and SD (*P*=0.327, and *P*=0.308, respectively) (Figure 2E). Some trends were observed for a relationship between therapy efficacy and relative changes of hematologic parameters, including LDH, NLR, PLR and SII pre- and post-treatments. With an increased sample size, the influence of LDH, NLR, PLR and SII in such patients would be further ascertained.

### Relationship between therapy efficacy and changes in levels of tumor markers

**Figure 3:**
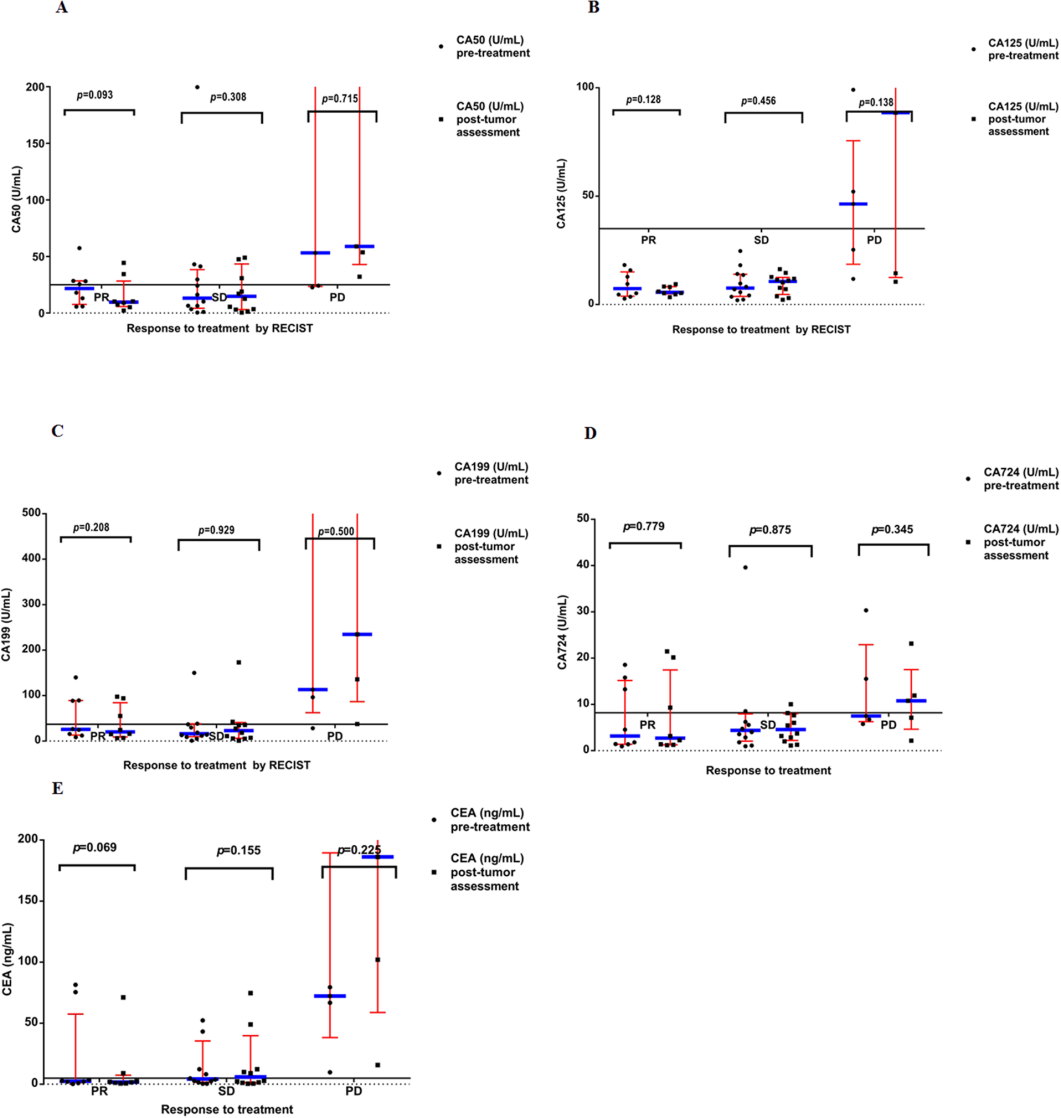
**(A)** Serum levels of CA50 pre-treatments and post-tumor assessment in the all patients enrolled on study. Results are divided in six dot plots, representative of the levels of CA50 pre-treatments versus post-tumor assessment in patients who showed PR, SD and PD. The horizontal line of X axis represents the upper limit of normal (ULN) value of CA50. **(B)** Serum levels of CA125 pre-treatments and post-tumor assessment in the all patients enrolled on study. Results are divided in six dot plots, representative of the levels of CA125 pre-treatments versus post-tumor assessment in patients who showed PR, SD and PD. The horizontal line of X axis represents the upper limit of normal (ULN) value of CA125. **(C)** Serum levels of CA199 pre-treatments and post-tumor assessment in the all patients enrolled on study. Results are divided in six dot plots, representative of the levels of CA199 pre-treatments versus post-tumor assessment in patients who showed PR, SD and PD. The horizontal line of X axis represents the upper limit of normal (ULN) value of CA199. **(D)** Serum levels of CA724 pre-treatments and post-tumor assessment in the all patients enrolled on study. Results are divided in six dot plots, representative of the levels of CA724 pre-treatments versus post-tumor assessment in patients who showed PR, SD and PD. The horizontal line of X axis represents the upper limit of normal (ULN) value of CA724. **(E)** Serum levels of CEA pre-treatments and post-tumor assessment in the all patients enrolled on study. Results are divided in six dot plots, representative of the levels of CEA pre-treatments versus post-tumor assessment in patients who showed PR, SD and PD. The horizontal line of X axis represents the upper limit of normal (ULN) value of CEA. *P* values are the result of the nonparametric Wilcoxon Signed Rank test. In all dot plots, the median and interquartile range are shown. RECIST, Response Evaluation Criteria in Solid Tumors; PR, partial response; SD, stable disease; PD, progressive disease. *P* values<0.05 are considered as statistically significant.

We correlated the change of tumor markers’ levels with anticancer response to treatment by RECIST criteria. The data from our study also showed that no obvious differences were observed in levels of CA50 before and after therapies among tumor sufferers with PR, SD and PD (*P*=0.093, *P*=0.308, and *P*=0.715, correspondingly) (Figure 3A), or in levels of CA125 before and after therapies among tumor sufferers with PR, SD and PD (*P*=0.128, *P*=0.456, and *P*=0.138, correspondingly) (Figure 3B), or in levels of CA199 before and after therapies among tumor sufferers with PR, SD and PD (*P*=0.208, *P*=0.929, and *P*=0.500, correspondingly) (Figure 3C), or in levels of CA724 before and after therapies among tumor sufferers with PR, SD and PD (*P*=0.779, *P*=0.875, and *P*=0.345, correspondingly) (Figure 3D), or in levels of CEA before and after therapies among tumor sufferers with PR, SD and PD (*P*=0.069, *P*=0.155, and *P*=0.225, correspondingly) (Figure 3E).

### Correlations of therapeutic response and clinical characteristics

A statistical analysis of the effect of age at the time of treatment demonstrated no age-related significant difference in the response to treatment. However, some tendencies were illustrated for an association between age and clinical response among groups (PR vs. SD vs. PD) (*P*=0.084). A more advanced age of patients did not negatively affect the therapeutic response. The distribution of gender during the treatment manifested no significant differences among groups (PR vs. SD vs. PD) (*P*=0.198). The statistical analysis demonstrated that the distribution of patient performance status following treatment did not differ significantly in correlation to the response to treatment (*P*=0.62). No statistical significance was found in the analysis of therapeutic response to the primary stage of disease (IVA vs. IVB vs. IVC) (*P*=0.883). Yet the statistical analysis failed to demonstrate differences in response to therapy with respect to primary location of the tumor in the ascending colon, transverse colon, descending colon, rectum or sigmoid colon (*P*=0.52). Statistically, the grade of histological malignancy could not be demonstrated to affect the response to treatment. No relationship was demonstrated by statistical analysis between different line of therapy and response to treatment (*P*=0.223). No obvious variations were noted between response to treatment and associated therapy (including FOLFOX6, FOLFIRI, chemotherapy plus bevacizumab, chemotherapy plus Cetuximab combination) (*P*=0.852). Statistically, no effect of smoking history, alcohol-drinking history or first-degree relative cancer history could be demonstrated on response to therapy (Table 2).

**Table 2:** Correlations of tumor response and clinical characteristics

Characteristic	Number	PR	SD	PD	*P* -Value
Total	25	8	12	5	
Age(years)					0.084
<60	13	2	9	2	
≧60	12	6	3	3	
Gender					0.198
Male	16	4	7	5	
Female	9	4	5	0	
ECOG PS					0.62
0	12	3	7	2	
1	13	5	5	3	
Stages					0.883
IVA	10	4	3	3	
IVB	10	3	6	1	
IVC	5	1	3	1	
Tumor localization					0.52
Ascending colon	6	1	4	1	
Transverse colon	1	1	0	0	
Descending colon	3	1	0	2	
Sigmoid colon	8	3	4	1	
Rectum	7	2	4	1	
Differentiation grade					0.615
1	1	0	1	0	
2	17	7	7	3	
3	7	1	4	2	
Line of therapy					0.223
First	9	5	2	2	
Second	10	2	7	1	
Third	6	1	3	2	
Associated therapy					0.852
FOLFOX6	4	2	2	0	
FOLFIRI	7	1	4	2	
CT+B	10	3	5	2	
CT+Cetuximab	4	2	1	1	
Smoking history					0.791
No	21	6	10	5	
Yes	4	2	2	0	
Alcohol-drinking history					1
No	22	7	10	5	
Yes	3	1	2	0	
First-degree relative cancer history					0.68
No	23	8	10	5	
Yes	2	0	2	0	

*P value ≤0.05 was considered statistically significant*.

*ECOG, Eastern Cooperative Oncology Group; PS, performance status; CT, chemotherapy B, bevacizumab; PR: Partial response; SD: Stable disease; PD: Progression disease*.

### Correlations of variable and therapeutic response

We found a statistically significant strong correlation between the percent of relative change in Tregs: CD4 ratio and therapeutic response (Spearman’s rho=0.788/*P*=0.000). The correlation was weak between the percentage of relative change in LDH and response to therapy (Spearman’s rho=0.359/*P*=0.039). Testing using Spearman’s rho yielded no statistically significant correlation between the percentage of relative change in NLR and therapeutic response (Spearman’s rho=0.267/*P*=0.098). On Spearman correlation testing, no correlation was found between age and response to therapy (Spearman’s rho=-0.085/*P*=0.344). Response's correlations were shown in Table 3.

**Table 3:** Response's correlations

Variable	Spearman's rho	Correlation *P* -value
% relative change in Treg: CD4 ratio	0.788	0.000
% rellative change in LDH	0.359	0.039
% rellative change in NRL	0.267	0.098
age	-0.085	0.344

Correlation is assumed to be significant at *P* = 0.05.

Treg, regulatory T Cells; LDH, lactate dehydrogenase; NLR, neutrophil-lymphocyte ratio.

## DISCUSSION

Tregs play a critical part in immune homeostasis owing to their capability of suppressing immoderate immune responses. Larger numbers of Tregs in peripheral blood, TDLNs, and neoplasm tissues have been noted in CRC patients [8, 20, 21]. As has been demonstrated, most of the tumor-resident Tregs are thymus derived, while about half of the Treg population in nontumoral areas of the colon is induced Tregs (iTregs) which are generated in the periphery [22]. However, the role of Tregs in CRC is debatable [23]. The higher FoxP3^+^ Treg frequencies in the tumor microenvironment are significantly correlated with advanced stage and poor prognosis of CRC [24]. What’s more, it has been revealed that Tregs can hinder CD4^+^ T cell responses to colorectal cancer antigens, thus contributing to malignant progression [8]. Meanwhile, higher percentages of intratumoral FoxP3^**+**^ cells are revealed to be correlated with a favorable outcome in colorectal cancer patients [25]. It has been indicated that the stage of the disease and the type of immune responses which are formed in the tumor microenvironment can be decisive. Tregs seem to reduce detrimental inflammation and control tumorigenesis in early stages of carcinogenesis, while at late stages they can promote tumor growth and dissemination through down-regulating anticancer immune responses [26].

It should be of note that currently most of the clinical studies use FoxP3 and CD25 as Treg markers. Even though CD25^high^ FoxP3^**+**^ T cells include substantial suppressive Treg populations, high levels of CD25 and FoxP3 expression can be induced on non-suppressive T cells in the process of activation or inflammation [27]. And yet, due to Foxp3’s expression in the nucleus of Tregs, permeabilization of cellular and nuclear membranes is needed. Accordingly, it cannot be utilized as an isolation marker for viable Tregs. By contrast, current evidence has revealed that CD127 is expressed lowly on the surface of Tregs [28]. In CD25^high^ Tregs, the lower expression level of CD127 exhibits more sufficient suppressive capacity and the correlative higher expression of Foxp3. And in spite of that, in the majority of clinical investigations, the suppressive function of Tregs was not appraised. Moreover, CD127 is not utilized as a negative marker for Treg, and not every FoxP3^**+**^ human Treg displays functionally suppressive property [29]. The combined utilization of biomarkers, including CD25^high^ and CD127^neg^, might better identify Treg populations with suppressive capacity [28]. As mentioned above, we would use the CD4^+^CD25^+^CD127^neg^ phenotype as Treg markers. Use of the CD4^+^CD25^+^CD127^neg^ phenotype to define Tregs may help users to better understand the role of Tregs in the majority of pathological conditions.

Considering all the current evidences, it is needless to say that PD1/PDL1 pathway and Tregs are vital to the maintenance of dynamic T cell homeostasis, and may involve in the same pathway. An important role has been suggested of PD-L1 in sustaining the suppressive properties of the Treg population. Blockade of PD1/PDL1 pathway also prevents the conversion of naive Th cells to Tregs [30]. Furthermore, PD-L1 can upregulate FOXP3 expression, thereby enhancing its suppressive function [31]. Initially, immune checkpoint inhibitors (ICPI) strategies are evolved to potentiate or rescue the effector functions of the antitumor cell-mediated immune responses. Nonetheless, it is becoming increasingly evident that ICPI may also exert an influence on some other components of immunity system, including Treg ablation. Recent studies show that tumor-infiltrating Tregs can induce much higher expression of many immune checkpoint molecules such as LAG-3, TIM-3, GITR, CTLA-4 and PD-1, making them feasible targets for ICPI [32]. For clinical medicines, the advent of ICPI, other than currently available treatments, offers valuable therapeutic modalities for Treg impairment or depletion. Targeting Tregs—probably as fraction of a multi-modal therapeutic approach—presents a promising therapeutic modality to restore the immune equilibrium and eliminate immunosuppression within the tumor microenvironment [32]. Thus, converting current Treg biological knowledge into practicable immunological therapy needs a deeper comprehension of the immune dynamics of Tregs in a large range of cancers, the interactions of Tregs with tumor-infiltrating cell subsets, and the influence of diverse oncologic therapies on Tregs *in vivo*.

Our study has confirmed recent preliminary clinical data [33] that advanced malignancy is often marked by an abnormal rise in Treg cell count and consequently a rise in the Treg/TH ratio, usually owing to both Treg rise and TH lymphocyte fall. Moreover, even though limited to a relatively low number of patients, it has clearly shown that the various classical anticancer therapies may induce marked variations of Treg generation, in terms of percentage with regard to the TH lymphocyte count. As far as the treatment is concerned, the influence of therapy on Treg generation claims special attention since it appears to be different in comparison to the clinical response. In fact, the Treg cell number declines in the patients with objective tumor regression or SD, whereas it increases in the patients who have PD. Moreover, a therapy-induced decrease in the Treg/TH ratio has been proven to anticipate the therapeutic efficacy, in terms of tumor growth control [33]. Similarly, Roselli et al has observed that the most of tumor sufferers show either a slight variation or a rise in the TH / Treg ratio in the treatment, as well as either a slight variation or a decline in Treg suppressive function in the treatment. It is also found that a correlation (*P*=0.036) appears between a decline in Treg frequency in the course of FOLFIRI treatment and overall survival (OS), and a correlation (*P*=0.037) between Treg frequency pre-treatment and PFS. Responders to the chemical therapy by RECIST criteria also reveal a pretty significant decline in Tregs during treatment vs. pre-treatment (*P*=0.0064) with regard to non-responders [34].

Although these findings reported here denote only a fraction of the multifaceted manifestation of host immune responses to conventional antitumor drugs, they indicate that not every chemotherapeutic agent is likely to exert an unfavorable influence on the immunologic system of tumor sufferers, while others either have no influence or may dampen down immunosuppressive pathways, including those mediated by Tregs [35]. Moreover, having in mind these immune responses, our findings suggest that some specific conventional antitumor treatments can potentially be combined with immuno-oncology drugs to improve antitumor clinical efficacy. Accordingly, they may inform clinicians on the rational design of future trials implementing conventional, standard-of-care treatments combined with vanguard immune-stimulatory modalities toward an ever more effective therapy of tumor sufferers [35].

However, recently used RECIST (responders vs. non-responders) parameters seem to be inadequate to document the differences in treatment response [36]. Conventional RECIST criteria have been interrogated as a reliable index of clinical endpoint in tumor sufferers treated with an anti-angiogenesis inhibitor, as these antiangiogenic drugs often induce tumor alterations independent of obvious changes in tumor measurements [37]. Thus a number of the divergences may be explained in the clinical significance of reduced Tregs as reported in the literature, which possibly will rely on the criteria of therapeutic reaction selected (OS, PFS, RECIST) [38]. As indicated by increasing clinical experience, traditional response criteria may not be sufficient to fully characterize activity in this new era of targeted therapies and/or biologics. For example, stable disease (SD) is characterized as either an increase or a decrease in tumor burden insufficient in magnitude to qualify as PD or PR, respectively. With chemotherapy, SD, often transient, is not considered indicative of true antitumor activity. In contrast, with tyrosine kinase inhibitors, maintaining SD has been identified as a conceivable surrogate endpoint for favorable clinical outcome (median time to progression). Interpretation of this endpoint under the RECIST criteria, therefore, has been revisited in recent years, and duration of limited regressions or extended SD attained by these molecules is, in some cases, now viewed as evidence of activity [39]. Although potentially an improvement over conventional criteria for immunotherapeutic agents, the immune-related response criteria (irRC) may still not capture or fully characterize all relevant patterns of clinical activity. For example, one challenge to the irRC is that the term “irSD” is appropriate both for cases of minimal change in tumor burden over time and for large increases in tumor burden followed by a reduction to baseline levels. In clinical practice, where the current tumor assessment is often compared with the most recent one (i.e. the baseline is “reset” to reflect the latest measurements), this latter case may be more appropriately characterized as an objective response and not as SD [39]. Accordingly, there is a need for more sensitive, specific, and reliable haematological parameters to monitor therapeutic response to such treatment. In particular, allowing for tumor sufferers receiving translational treatment, an easy and rapid approach to evaluate antitumor efficacy after only 4 weeks (two cycles) would make allowance for a timely conversion in clinical regimens to accomplish resection of hepatic metastases and minimize hepatic toxicity of successive therapy schedules [40].

Apparently, the relatively low number of patients appraised in this study in relation to the various anticancer treatments does not allow more definite conclusions. Nevertheless, in virtue of these preliminary results, the Treg cell’s percentage with regard to TH cell number could represent key immune parameters for monitoring the clinical course of a malignant disease. Hence, these results further justify longitudinal studies to better establish the influence of classical anticancer treatments on Treg production and its possible prognostic significance. To a considerable extent, our findings, if confirmed in larger, prospective series, may represent an important asset for both clinical practice and clinical trial design. The opportunity to use a widely available, easy-to-obtain set of circulating haematological parameters, could in fact bring clinicians on the one hand to an early identification of patients responding to therapy and on the other hand to an early identification of clinically resistant patients, who potentially constitute candidates for more aggressive treatments [41]. In the era of novel biological markers and molecularly targeted agents, information deriving from clinical assessment and circulating haematological parameters that are already available in the clinic could help in composing the complex scenario of CRC.

In conclusion, to our knowledge, the results of the current study demonstrate that for the first time, the monitoring of the relative change in Treg/TH ratio may well offer a promising predictive parameter for treatment efficacy of anticancer therapies. As such, it is expected that these parameters will have a broad applicability to immunotherapeutic agents and molecularly targeted agents.

## MATERIALS AND METHODS

### Study population and data collection

A total of 25 mCRC patients undergoing classical anticancer therapies were enrolled in the study at the First Affiliated Hospital of Xiamen University. Patients were recruited from December 2015 to August 2016 and followed up until December 2016. The inclusion criteria were: a histologic diagnosis of stage IV colorectal carcinoma; Eastern Cooperative Oncology Group performance status of 0–2; at least one measurable lesion according to the Response Evaluation Criteria in Solid Tumors [RECIST]; no limit of age and life expectancy; and adequate organ function. Exclusion criteria were as follows: any major organ failure, central nervous system involvement, metachronous malignancy, severe drug hypersensitivity, active infectious diseases, major autoimmune diseases, and acquired immune suppression. The study protocol was approved by the Human and Animal Ethics Review Committee of the Medical college of Xiamen University, China, and a signed consent form was obtained from each patient before we collected the blood samples used in this study.

A series of baseline clinical variables were collected from patients’ medical records as follows: patient demographics, smoking and drinking history, family history of cancer, date of diagnosis and some tumor characteristics, such as tumor location, differentiation grade and tumor stage. The histopathological findings were classified according to the World Health Organization criteria, and patients were staged according to the 8th edition of American Joint Committee on Cancer (AJCC) TNM classification system. The relation of the Eastern Cooperative Oncology Group (ECOG) performance status was also evaluated. Moreover, hematologic parameters were collected, including serum albumin, hemoglobin, lymphocytes and platelets. Then, the systemic immune-inflammation index (SII) was calculated as platelet count × neutrophil count/lymphocyte count, NLR was obtained by dividing the absolute neutrophil count by the absolute lymphocyte count, and PLR was calculated as the ratio of absolute platelet count to absolute lymphocyte count. Serum levels of CA50, CA125, CA199, CA724 and CEA were considered normal at levels between 0–25 U/ml, 0–35 U/ml, 0–37 U/ml, 0–8.2 U/ml and 0–5 ng/ml, respectively. Lymphocytes, platelets and neutrophils were evaluated by hemocytometric cell counts, while their feature was evaluated by microscope analysis. Whole blood of mCRC patients was sampled before (D0) and after anticancer therapies (D30) (post-tumor assessment). All analyses were performed following the first 6 hours after sampling. The raw data regarding clinical, pathologic, biochemical, haematological parameters and tumor biomarkers of patients were presented in Supplementary Tables 1-6. We calculated the relative change in LDH for each patient as follows: (value of LDH post-tumor assessment - value of LDH pre-treatments) / value of LDH pre-treatments. Review of pathology reports confirmed the diagnosis. Information regarding clinical, pathologic, and biological characters of patients was presented in Table 1.

### Treatment schedules and patients’ evaluation

All mCRC patients were diagnosed in our cancer center and were proposed to be treated by FOLFOX–based (oxaliplatin, levo-leucovorin, 5-fluorouracil (5-FU)) or FOLFIRI (irinotecan, levo-leucovorin, 5-fluorouracil (5-FU)) chemotherapy alone or plus bevacizumab or Cetuximab combination as first-line or second or third line regimen. Standard assessments (clinical history, physical examination, hematochemical analysis, Tumor Marker assays, contrast enhanced thorax-abdomen pelvis CT scan or MRI and bone scintigraphy) were performed within 10 days before classical anticancer therapy began, and repeated after 30 days. Blood was always retrieved before administration of anticancer therapy. Response to therapy was evaluated by computed tomography or magnetic resonance imaging according to the RECIST by a trained radiologist and defined in categories of progressive disease, stable disease, or partial and complete remission. Early changes in immune cells, after 1 month of therapy, were correlated with the best radiologic response in accordance with RECIST criteria.

### Measurement of regulatory T cells

For the immune evaluation, the venous blood samples were collected in the morning after an overnight fast. After Ficoll (Sigma) separation, human peripheral blood mononuclear cells were resuspended in staining buffer (PBS containing 3% fetal bovine serum) and stained for 30 min at 4°C with FITC-conjugated anti-CD4 (Beckman Coulter), PCy5-conjugated anti-CD25 (Beckman Coulter) and PE-conjugated anti-CD127 (Beckman Coulter). Flow cytometry was performed on a FC500 flow cytometer (Beckman Coulter): 5×10^4^ events were acquired and data were analyzed using CXP software (Beckman Coulter). To determine the percentage of Tregs, lymphocytes were gated by plotting forward versus side scatter. The CD4^+^ population was gated first, followed by the CD25^+^CD127^neg^ population. Tregs are thus defined as the CD4^+^CD25^+^CD127^neg^ population. In each blood sample the T helper lymphocytes (CD4^+^), Treg lymphocytes (CD4^+^CD25^+^CD127^neg^) and the Treg/TH ratio (CD4^+^CD25^+^CD127^neg^ / CD4^+^) were evaluated. The CD4^+^ and CD4^+^CD25^+^CD127^neg^ cells were measured by flow cytometric analysis. The normal values obtained in our laboratory (95% confidence limits) were lower than 5% for the Treg/TH ratio. We calculated the relative change in the ratio of Treg/TH for each patient as follows: (value of the ratio of Treg/TH post-tumor assessment - value of the ratio of Treg/TH pre-treatments) / (value of the ratio of Treg/TH pre-treatments).

### Statistical analysis

Parametric data were expressed as mean ±SD; non-parametric data were expressed as median and interquartile ranges (IQR); whereas categorical data were reported as proportions. For the analysis of data, the nonparametric Wilcoxon Signed Rank test was used to compare continuous data for paired samples, or the Kruskal–Wallis test was used for multiple comparisons; whereas the Chi-square test or Fisher exact test (if expected frequencies were≦5) was applied for categorical variables, as appropriate. Spearman’s rank correlation coefficient(r) (–1.00 to +1.00) was used to calculate the correlation between two variables. The strength of the correlation was considered as perfect if ρ= 1; strong if 0.7≦ρ<1; moderate if 0.5≦ρ< 0.7; weak if 0.3≦ρ<0.5; and no relationship if ρ<0.3, as our aim was to evaluate the positive correlation between response to treatment and the relative change in the ratio of Treg/TH. Statistical significance was assumed at a *p*-value of 0.05. All statistical analyses were performed by using SPSS version 24.0 (IBM Corporation, Armonk, NY, USA). Graphs were prepared using GraphPad Prism 7 software (GraphPad Software, Inc., USA).

## SUPPLEMENTARY MATERIALS TABLES



## References

[1] Torre LA, Bray F, Siegel RL, Ferlay J, Lortet-Tieluent J, Jemal A (2015). Global cancer statistics, 2012. CA Cancer J Clin.

[2] Ménétrier-Caux C, Curiel T, Faget J, Manuel M, Caux C, Zou W (2012). Targeting regulatory T cells. Target Oncol.

[3] deLeeuw RJ, Kost SE, Kakal JA, Nelson BH (2012). The prognostic value of FoxP3+ tumor-infiltrating lymphocytes in cancer: a critical review of the literature. Clin Cancer Res.

[4] Finotello F, Trajanoski Z (2017). New strategies for cancer immunotherapy: targeting regulatory T cells. Genome Med.

[5] Nishikawa H, Sakaguchi S (2014). Regulatory T cells in cancer immunotherapy. Curr Opin Immunol.

[6] Wang K, Vella AT (2016). Regulatory T cells and cancer: a two-sided story. Immunol Invest.

[7] Tanaka A, Sakaguchi S (2017). Regulatory T cells in cancer immunotherapy. Cell Res.

[8] Betts G, Jones E, Junaid S, El-Shanawany T, Scurr M, Mizen P, Kumar M, Jones S, Rees B, Williams G, Gallimore A, Godkin A (2012). Suppression of tumour-specific CD4+ T cells by regulatory T cells is associated with progression of human colorectal cancer. Gut.

[9] Clarke SL, Betts GJ, Plant A, Wright KL, El-Shanawany TM, Harrop R, Torkington J, Rees BI, Williams GT, Gallimore AM, Godkin AJ (2006). CD4+CD25+FOXP3+ regulatory T cells suppress anticancer immune responses in patients with colorectal cancer. PLoS One.

[10] Tan W, Zhang W, Strasner A, Grivennikov S, Cheng JQ, Hoffman RM, Karin M (2011). Tumour-infiltrating regulatory T cells stimulate mammary cancer metastasis through RANKL-RANK signalling. Nature.

[11] Sierzega M, Lenart M, Rutkowska M, Surman M, Mytar B, Matyja A, Siedlar M, Kulig J (2017). Preoperative neutrophil-lymphocyte and lymphocyte-monocyte ratios reflect immune cell population rearrangement in resectable pancreatic cancer. Ann Surg Oncol.

[12] Hu B, Yang XR, Xu Y, Sun YF, Sun C, Guo W, Zhang X, Wang WM, Qiu SJ, Zhou J, Fan J (2014). Systemic immune-inflammation index predicts prognosis of patients after curative resection for hepatocellular carcinoma. Clin Cancer Res.

[13] Hong X, Cui B, Wang M, Yang Z, Wang L, Xu Q (2015). Systemic immune-inflammation index, based on platelet counts and neutrophil-lymphocyte ratio, is useful for predicting prognosis in small cell lung cancer. Tohoku J Exp Med.

[14] Passardi A, Scarpi E, Cavanna L, Dall’Agata M, Tassinari D, Leo S, Bernardini I, Gelsomino F, Tamberi S, Brandes AA, Tenti E, Vespignani R, Frassineti GL (2016). Inflammatory indexes as predictors of prognosis and bevacizumab efficacy in patients with metastatic colorectal cancer. Oncotarget.

[15] Li MX, Liu XM, Zhang XF, Zhang JF, Wang WL, Zhu Y, Dong J, Cheng JW, Liu ZW, Ma L, Lv Y (2014). Prognostic role of neutrophil-to-lymphocyte ratio in colorectal cancer: a systematic review and meta-analysis. Int J Cancer.

[16] Templeton AJ, McNamara MG, Šeruga B, Vera-Badillo FE, Aneja P, Ocaña A, Leibowitz-Amit R, Sonpavde G, Knox JJ, Tran B, Tannock IF, Amir E (2014). Prognostic role of neutrophil-to-lymphocyte ratio in solid tumors: a systematic review and meta-analysis. J Natl Cancer Inst.

[17] Krauthamer M, Rouvinov K, Ariad S, Man S, Walfish S, Pinsk I, Sztarker I, Charkovsky T, Lavrenkov K (2013). A study of inflammation-based predictors of tumor response to neoadjuvant chemoradiotherapy for locally advanced rectal cancer. Oncology.

[18] Kim IY, You SH, Kim YW (2014). Neutrophil-lymphocyte ratio predicts pathologic tumor response and survival after preoperative chemoradiation for rectal cancer. BMC Surg.

[19] Wu Y, Li C, Zhao J, Yang L, Liu F, Zheng H, Wang Z, Xu Y (2016). Neutrophil-to-lymphocyte and platelet-to-lymphocyte ratios predict chemotherapy outcomes and prognosis in patients with colorectal cancer and synchronous liver metastasis. World J Surg Oncol.

[20] Bacić D, Uravić M, Bacić R, Sutić I, Petrosić N (2011). Augmentation of regulatory T cells (CD4+CD25+Foxp3+) correlates with tumor stage in patients with colorectal cancer. Coll Antropol.

[21] Shimabukuro-Vornhagen A, Schlößer HA, Gryschok L, Malcher J, Wennhold K, Garcia-Marquez M, Herbold T, Neuhaus LS, Becker HJ, Fiedler A, Scherwitz P, Koslowsky T, Hake R (2014). Characterization of tumor-associated B-cell subsets in patients with colorectal cancer. Oncotarget.

[22] Jafarinia M, Mehdipour F, Hosseini SV, Ghahramani L, Hosseinzadeh M, Ghaderi A (2016). Determination of a CD4^+^CD25^-^FoxP3^+^ T cells subset in tumor-draining lymph nodes of colorectal cancer secreting IL-2 and IFN-γ. Tumour Biol.

[23] Zhang X, Kelaria S, Kerstetter J, Wang J (2015). The functional and prognostic implications of regulatory T cells in colorectal carcinoma. J Gastrointest Oncol.

[24] Chaput N, Louafi S, Bardier A, Charlotte F, Vaillant JC, Ménégaux F, Rosenzwajg M, Lemoine F, Klatzmann D, Taieb J (2009). Identification of CD8+CD25+Foxp3+ suppressive T cells in colorectal cancer tissue. Gut.

[25] Mougiakakos D (2011). Regulatory T cells in colorectal cancer: from biology to prognostic relevance. Cancers (Basel).

[26] Whiteside TL (2012). What are regulatory T cells (Treg) regulating in cancer and why?. Semin Cancer Biol.

[27] Gavin MA, Torgerson TR, Houston E, DeRoos P, Ho WY, Stray-Pedersen A, Ocheltree EL, Greenberg PD, Ochs HD, Rudensky AY (2006). Single-cell analysis of normal and FOXP3-mutant human T cells: FOXP3 expression without regulatory T cell development. Proc Natl Acad Sci U S A.

[28] Shen LS, Wang J, Shen DF, Yuan XL, Dong P, Li MX, Xue J, Zhang FM, Ge HL, Xu D (2009). CD4(+)CD25(+)CD127(low/-) regulatory T cells express Foxp3 and suppress effector T cell proliferation and contribute to gastric cancers progression. Clin Immunol.

[29] Wang J, Ioan-Facsinay A, van der Voort EI, Huizinga TW, Toes RE (2007). Transient expression of FOXP3 in human activated nonregulatory CD4+ T cells. Eur J Immunol.

[30] Burton OT, Zaccone P, Phillips JM, De La Peña H, Fehérvári Z, Azuma M, Gibbs S, Stockinger B, Cooke A (2010). Roles for TGF-beta and programmed cell death 1 ligand 1 in regulatory T cell expansion and diabetes suppression by zymosan in nonobese diabetic mice. J Immunol.

[31] Jin HT, Ahmed R, Okazaki T (2011). Role of PD-1 in regulating T-cell immunity. Curr Top Microbiol Immunol.

[32] Chaudhary B, Elkord E (2016). Regulatory T cells in the tumor microenvironment and cancer progression: role and therapeutic targeting. Vaccines (Basel).

[33] Lissoni P, Brivio F, Fumagalli L, Messina G, Meregalli S, Porro G, Rovelli F, Vigorè L, Tisi E, D'Amico G (2009). Effects of the conventional antitumor therapies surgery, chemotherapy, radiotherapy and immunotherapy on regulatory T lymphocytes in cancer patients. Anticancer Res.

[34] Roselli M, Formica V, Cereda V, Jochems C, Richards J, Grenga I, Orlandi A, Ferroni P, Guadagni F, Schlom J (2016). The association of clinical outcome and peripheral T-cell subsets in metastatic colorectal cancer patients receiving first-line FOLFIRI plus bevacizumab therapy. Oncoimmunology.

[35] Roselli M, Cereda V, di Bari MG, Formica V, Spila A, Jochems C, Farsaci B, Donahue R, Gulley JL, Schlom J, Guadagni F (2013). Effects of conventional therapeutic interventions on the number and function of regulatory T cells. Oncoimmunology.

[36] Trillet-Lenoir V, Freyer G, Kaemmerlen P, Fond A, Pellet O, Lombard-Bohas C, Gaudin JL, Lledo G, Mackiewicz R, Gouttebel MC, Moindrot H, Boyer JD, Chassignol L (2002). Assessment of tumour response to chemotherapy for metastatic colorectal cancer: accuracy of the RECIST criteria. Br J Radiol.

[37] Adotevi O, Pere H, Ravel P, Haicheur N, Badoual C, Merillon N, Medioni J, Peyrard S, Roncelin S, Verkarre V, Mejean A, Fridman WH, Oudard S, Tartour E (2010). A decrease of regulatory T cells correlates with overall survival after sunitinib-based antiangiogenic therapy in metastatic renal cancer patients. J Immunother.

[38] Finke JH, Rini B, Ireland J, Rayman P, Richmond A, Golshayan A, Wood L, Elson P, Garcia J, Dreicer R, Bukowski R (2008). Sunitinib reverses type-1 immune suppression and decreases T-regulatory cells in renal cell carcinoma patients. Clin Cancer Res.

[39] Wolchok JD, Hoos A, O'Day S, Weber JS, Hamid O, Lebbé C, Maio M, Binder M, Bohnsack O, Nichol G, Humphrey R, Hodi FS (2009). Guidelines for the evaluation of immune therapy activity in solid tumors: immune-related response criteria. Clin Cancer Res.

[40] Schauer D, Starlinger P, Alidzanovic L, Zajc P, Maier T, Feldman A, Padickakudy R, Buchberger E, Elleder V, Spittler A, Stift J, Pop L, Gruenberger B (2016). Chemotherapy of colorectal liver metastases induces a rapid rise in intermediate blood monocytes which predicts treatment response. Oncoimmunology.

[41] Masi G, Loupakis F, Salvatore L, Fornaro L, Cremolini C, Cupini S, Ciarlo A, Del Monte F, Cortesi E, Amoroso D, Granetto C, Fontanini G, Sensi E (2010). Bevacizumab with FOLFOXIRI (irinotecan, oxaliplatin, fluorouracil, and folinate) as first-line treatment for metastatic colorectal cancer: a phase 2 trial. Lancet Oncol.

