# Supplementary material for: The relative change in regulatory T cells / T helper lymphocytes ratio as parameter for prediction of therapy efficacy in metastatic colorectal cancer patients
Source: Oncotarget. 2017 Nov 21;8(65):109079–93. doi: 10.18632/oncotarget.22606 (PMC5752505; doi:10.18632/oncotarget.22606)
Supplement: Supplementary file 1 [file oncotarget-08-109079-s001.pdf]

The relative change in regulatory T cells / T helper lymphocytes ratio as parameter for prediction of therapy efficacy in metastatic colorectal cancer patients

SUPPLEMENTARY MATERIALS

Supplementary Table 1: The raw data regarding clinical characters of patients

Number	Response	Gender	Age	Tumor localization	Differentiation grade	ECOG PS	Smoking history
1	PR	Female	66	Sigmoid colon	2	1	No
2	PR	Male	64	Descending colon	2	1	Yes
3	PR	Female	33	Sigmoid colon	2	0	No
4	PR	Male	62	Sigmoid colon	3	1	No
5	PR	Female	38	Rectum	2	0	No
6	PR	Male	68	Transverse colon	2	0	Yes
7	PR	Male	68	Rectum	2	1	No
8	PR	Female	74	Ascending colon	2	1	No
9	SD	Male	70	Rectum	2	1	No
10	SD	Female	61	Rectum	3	0	No
11	SD	Male	43	Ascending colon	2	0	No
12	SD	Female	55	Ascending colon	1	0	No
13	SD	Male	46	Sigmoid colon	3	1	No
14	SD	Male	56	Rectum	2	1	Yes
15	SD	Male	70	Rectum	2	0	No
16	SD	Male	57	Sigmoid colon	3	1	Yes
17	SD	Female	57	Ascending colon	3	0	No
18	SD	Male	54	Sigmoid colon	2	0	No
19	SD	Female	45	Sigmoid colon	2	0	No
20	SD	Female	59	Ascending colon	2	1	No
21	PD	Male	41	Sigmoid colon	2	0	No
22	PD	Male	62	Rectum	2	0	No
23	PD	Male	72	Ascending colon	2	1	No
24	PD	Male	58	Descending colon	3	1	No
25	PD	Male	65	Descending colon	3	1	No

Supplementary Table 2: The raw data regarding clinical and pathologic characters of patients

Number	Alcohol-drinking history	First-degree relative cancer history	KRAS status	Stages
1	No	No	Wild type	IVA
2	No	No	Missing	IVB
3	No	No	Missing	IVA
4	No	No	Missing	IVC
5	No	No	Missing	IVA
6	Yes	No	Missing	IVB
7	No	No	Wild type	IVB
8	No	No	Mutated	IVA
9	No	No	Mutated	IVA
10	No	No	Missing	IVC
11	No	No	Mutated	IVC
12	No	No	Wild type	IVB
13	No	No	Missing	IVB
14	Yes	Yes	Missing	IVB
15	No	No	Missing	IVA
16	Yes	Yes	Wild type	IVA
17	No	No	Missing	IVB
18	No	No	Missing	IVB
19	No	No	Wild type	IVC
20	No	No	Missing	IVB
21	No	No	Missing	IVB
22	No	No	Mutated	IVA
23	No	No	Wild type	IVA
24	No	No	Missing	IVC
25	No	No	Mutated	IVA

Supplementary Table 3: The raw data regarding clinical, biochemical and haematological parameters of patients

Number	Associated chemotherapy	Line of therapy	Neutrophils	Lymphocytes	Platelets	Hb	Albumin	LDH
1	CT+Cetuximab	First	2.89	1.21	338	101	40	302
2	CT+B	Second	2.95	1.3	214	123	42.9	413
3	CT+B	First	1.32	1.27	282	116	46.3	169
4	FOLFIRI	Second	3.76	1.83	263	118	39.9	345
5	FOLFOX6	First	5.98	0.9	560	114	34.7	533
6	FOLFOX6	First	1.62	1.9	157	146	46.8	150
7	CT+Cetuximab	Third	2.13	0.87	110	131	40.6	248
8	CT+B	First	5.4	1.56	448	98	38.5	335
9	CT+B	Second	2.59	1.03	280	128	45.2	246
10	FOLFOX6	First	1.99	0.38	149	119	43.8	166
11	CT+B	Third	2.35	0.76	160	157	45	599
12	CT+B	Third	3.52	1.35	243	112	40.9	251
13	FOLFIRI	Second	3.96	1.97	167	150	43	226
14	FOLFIRI	Second	2.55	1.5	326	141	50.3	213
15	FOLFIRI	Second	3.75	0.94	235	127	44	218
16	FOLFOX6	Third	3.03	1.33	164	146	48.1	171
17	CT+B	Second	5.64	1.5	179	126	44.3	765
18	FOLFIRI	First	5.43	2.83	494	124	45	200
19	CT+Cetuximab	Second	2.8	1.34	271	147	48.5	161
20	CT+B	Second	1.28	1.61	314	113	39.2	1722
21	CT+B	Third	4.18	1.13	211	150	47.7	131
22	CT+B	Third	2.59	2.69	271	143	45.2	411
23	CT+Cetuximab	First	4.02	0.95	298	114	43.1	471
24	FOLFIRI	Second	4.8	1.29	169	126	45.8	495
25	FOLFIRI	First	4.5	2.33	304	126	45.4	654

Neutrophils$\times 10^3/mm^3$;Neutrophils$\times 10^3/mm^3$;Platelets$\times 10^3/mm^3$;Hb(g/L);Albumin(g/L);LDH(U/L)

Supplementary Table 4: The raw data regarding tumor biomarkers and Treg/TH ratio levels of patients

Number	ALP	CA50	CA125	CA199	CA724	CEA	Treg/ TH(Pre)	Treg/ TH(Post)
1	87	18	12.8	26.51	15.8	75.45	8.3	6.1
2	77	25.47	3.6	15.65	1.44	0.98	7	6.1
3	50	13.12	4.6	24.76	0.95	2.17	8.5	5.9
4	58	28.17	2.7	139.9	18.55	2.27	5.8	4.5
5	123	5.88	15.8	8.32	1.82	0.25	9.5	7.5
6	127	28.46	18.2	89.32	1.39	81.48	6.7	5.4
7	130	57.39	5.2	88.61	4.54	2.71	11.4	7.8
8	116	5.69	9.5	12.25	13.26	3.67	5.3	3.1
9	208	199.67	7	1404.8	6.24	43.18	7.1	6.5
10	54	10.22	2.3	13.84	3.57	0.5	13.5	10.2
11	106	43.06	18.1	150.02	8.54	52.33	9	8.9
12	112	29.69	9.7	38.21	4.76	4.73	7.9	5.4
13	104	1.01	3.6	1	1.81	2.62	7.3	7.2
14	67	24.2	8.2	29.66	2.87	3.83	5.7	4.7
15	67	3.49	5.7	11.59	1.12	0.5	10.1	10.6
16	97	41.3	14.1	36.8	0.96	12.31	7.8	7.3
17	64	16.24	4.2	18.57	4.03	8.21	5.3	5.1
18	378	6.5	24.7	8.87	39.62	2.47	8.2	7.3
19	88	6.39	2	12.94	5.57	1.42	6.2	4.7
20	341	0.5	13.6	5.11	1249	396.19	8.3	7.5
21	89	53.18	11.8	113.1	6.76	9.78	5.8	8.3
22	132	483.76	25.3	4095.1	30.33	299.63	4.7	7
23	115	24.25	99.1	28.18	7.5	72.26	3.6	8.4
24	106	22.73	46.4	96.25	5.78	66.75	4	8
25	187	500	52.1	2705	15.52	79.4	4.4	6.2

ALP (U/L); CA50, CA125, CA199, CA724(U/ml);CEA(ng/ml); Treg/TH ratio(%)

Supplementary Table 5: The raw data regarding biochemical and haematological parameters of patients

Number	Hb(post)	Neutrophils(post)	Lymphocytes(post)	Platelets(post)	Albumin(post)	LDH(post)	ALP(post)
1	109	2.35	1.64	213	40.8	289	88
2	109	2.16	0.9	203	43.1	365	57
3	119	1.89	0.78	202	49.9	170	50
4	109	2.52	1.43	243	42.2	332	62
5	123	4.44	0.66	169	40.5	413	226
6	130	2.83	2.3	121	44.2	156	121
7	123	1.72	0.68	122	37.3	232	126
8	103	7.54	1.82	230	41.8	461	140
9	124	1.88	1.06	282	44.4	214	186
10	117	2.02	0.39	98	44.1	206	54
11	157	2.9	0.94	141	45.5	470	114
12	112	4.12	1.5	171	43.3	381	107
13	154	3	1.73	180	42.5	183	116
14	135	4.31	1.38	250	48.1	193	62
15	130	5.52	0.77	250	42.3	215	85
16	148	4.84	1.99	167	48.9	204	134
17	130	2.32	1.38	176	42.1	518	64
18	129	4.14	2.29	380	46.3	140	235
19	144	3	1.72	234	50.6	178	96
20	114	3.26	2.21	245	43.9	1316	244
21	155	4.9	1.18	179	48.1	140	109
22	147	3.86	2.1	232	45	487	137
23	113	4.09	0.72	277	43.8	888	149
24	105	12.05	1.11	267	39.6	884	197
25	126	4.87	1.77	328	43.1	900	252

Neutrophils×10^3/mm^3;Neutrophils×10^3/mm^3;Platelets×10^3/mm^3;Hb(g/L);Albumin(g/L);LDH(U/L);ALP(U/L)

Supplementary Table 6: The raw data regarding tumor biomarkers of patients

Number	CA50(post)	CA125(post)	CA199(post)	CA724(post)	CEA(post)
1	2.27	8.3	6.8	21.47	8.99
2	9.26	3.4	15.06	1.27	0.62
3	10.24	5.3	24.38	1.2	1.92
4	34.39	4.7	94.1	20.16	2.39
5	7.43	8.1	15.8	1.38	0.76
6	10.14	9.4	55.19	2.28	71.11
7	44.4	5.2	97.55	3.17	1.62
8	5.18	6	6.4	9.3	1.27
9	212.05	7.7	1280.57	10.03	48.96
10	12.54	7.1	10.82	6.02	0.5
11	49.02	14.6	173.07	7.66	74.65
12	30.83	12.7	42.44	5.44	8.96
13	0.5	3.8	1	1.29	2.22
14	17.1	11.9	27.49	2.01	2.88
15	3.43	10.2	7.25	2.86	1.16
16	47.6	16.3	35.4	3.17	9.99
17	18.98	3	34.73	8.13	12.27
18	3.1	11.1	5.25	1.12	0.52
19	5.52	2.2	18.2	3.72	1.58
20	1.225	11.4	5.7	754.8	449.79
21	53.67	14.4	135.8	7.14	15.66
22	279.3	10.5	3400	23.14	261.3
23	32.16	225.5	37.56	11.92	102
24	58.9	238.8	234.7	2.17	303.86
25	500	88.5	2859	10.78	186.26

CA50, CA125, CA199, CA724(U/ml);CEA(ng/ml)
